# Absence of the Uterus

**Published:** 1842-03

**Authors:** 


*Absence of the Uterus.* By. Dr. Bertani.—Caroline Fossati, twenty years of age, when on the point of marriage, came to the hospital at Nular, to be treated for amenorrhœa. It was then ascertained that she had never menstruated, nor ever had had bloody discharge from any part of her body, which could supply the place of that secretion. Upon examination, the labia and nymphæ were found to be well formed. The clitoris was somewhat small. The urethra was in its natural situation, and of its ordinary shape. Below it, the carunculæ were in the form of an ellipsis, and appeared to surround the orifice of the vagina. On separating them, the mucous membrane was seen to be continuous, and not dilatable, scarcely yielding when a sound was pushed against it. Upon introducing a finger into the rectum, and an instrument into the bladder, they were found to be separated by no great thickness of parts. On examining all sides of the bladder with the sound, and depressing with the finger the abdominal parieties, no trace of a uterus or vagina could be detected.

The carriage and voice of Fossati were somewhat masculine. Her breasts began to appear at thirteen. There was hair on the pubes at fourteen; and at fifteen she had some abdominal pain in the loins and hypogastrium, which returned every month, but never with any discharge. Upon being told that she could not marry, she appeared not to suffer any grief. She had no sexual appetite. In other respects her tastes and desires were feminine.—*Jour. de Med. et de Chirurg. Prat.*, Nov. 1841, from *Annali Univers. di Med.* 1841.

